# Hydroxyurea-induced Tongue Hypermelanosis and Transverse Melanonychia

**DOI:** 10.7759/cureus.6311

**Published:** 2019-12-06

**Authors:** Elvira Neculiseanu, Janine Harewood, Gurinder Sidhu

**Affiliations:** 1 Medicine, State University of New York (SUNY) Downstate Medical Center, Brooklyn, USA

**Keywords:** hydroxyurea, hypermelanosis, mucocutaneous toxicity

## Abstract

Hydroxyurea (HU) is a commonly used medication for myeloproliferative neoplasm (MPN) and is usually well tolerated. Cutaneous toxicity of HU is well known and can be seen in several manifestations. We report a case of a man with MPL gene mutation associated with essential thrombocytosis, who had a rare mucocutaneous toxicity with diffuse tongue, skin and nail discoloration. Mucocutaneous toxicity is usually a benign condition and self-resolves after discontinuation of the medication. It can lead to patient anxiety and medication discontinuation. The mechanism for development of HU-induced mucocutaneous hyperpigmentation is poorly understood.

## Introduction

Hydroxyurea (HU) is a well-tolerated oral chemotherapeutic drug frequently used in the treatment of myeloproliferative neoplasms (MPNs), including essential thrombocytosis (ET). HU exerts its antitumor activity by inhibiting the enzyme ribonucleotide reductase and thereby inhibiting DNA synthesis [1]. While HU is a well-tolerated agent, it is known to have skin toxicity such as skin ulceration, oral aphthosis and mucocutaneous hyperpigmentation. There have been case reports of nail hyperpigmentation and tongue hyperpigmentation, but having both is rare [2,3]. We report a unique case with development of diffuse tongue hyperpigmentation, nail hyperpigmentation in all nails, knuckle hyperpigmentation, and palmar and plantar hyperpigmentation soon after the initiation of HU for the treatment of ET.

## Case presentation

**Figure 1:**
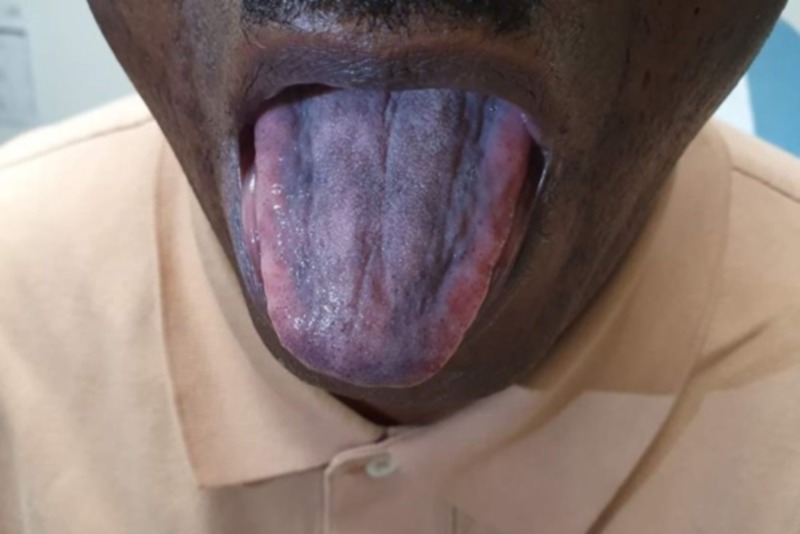
Hyperpigmentation of the tongue.

A 61-year-old African American man with past medical history of hypertension, diabetes mellitus and osteoarthritis was noted to have persistent isolated thrombocytosis on routine laboratory testing. He had no lymphadenopathy or hepatosplenomegaly. Laboratory testing showed a white blood cell count of 6.2 K/µL, hemoglobin 12 gm/dL, mean corpuscular volume 80, red cell distribution width 15 and platelet count of 1,197 K/µL. He underwent a bone marrow aspiration and biopsy which showed a hypercellularity for his age. Trilinear hematopoiesis was present with myeloid and erythroid maturation and increase in megakaryocytes noted, with some clustering. Molecular testing showed MPL exon 10 mutation was present; no mutation noted in BCR/ABL1, JAK2, and CALR; and a diagnosis of ET was made. The patient was started with HU 1,500 mg once daily and aspirin 81 mg. He tolerated the medications well and in three months his platelet count normalized. At that visit, the patient complained that he had developed diffuse tongue bluish/back discoloration (Figure 1).

**Figure 2:**
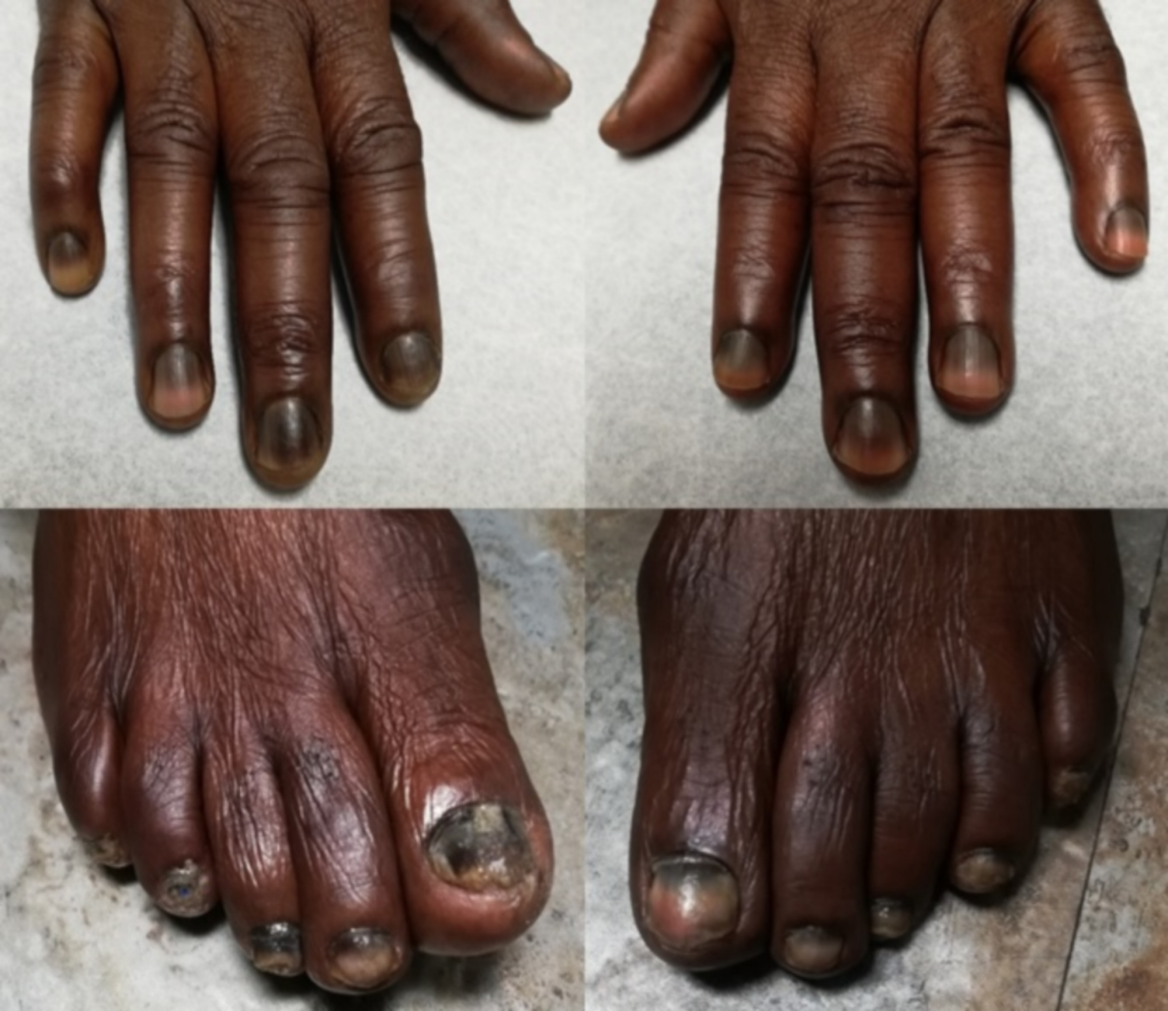
Hyperpigmentation of the skin over the knuckles, and transverse hyperpigmentation of all the nails.

There was no thickening, ulceration or tenderness in the tongue. There was no dysgeusia. He had also noted transverse bluish black discoloration of all his nails (Figure 2). 

**Figure 3:**
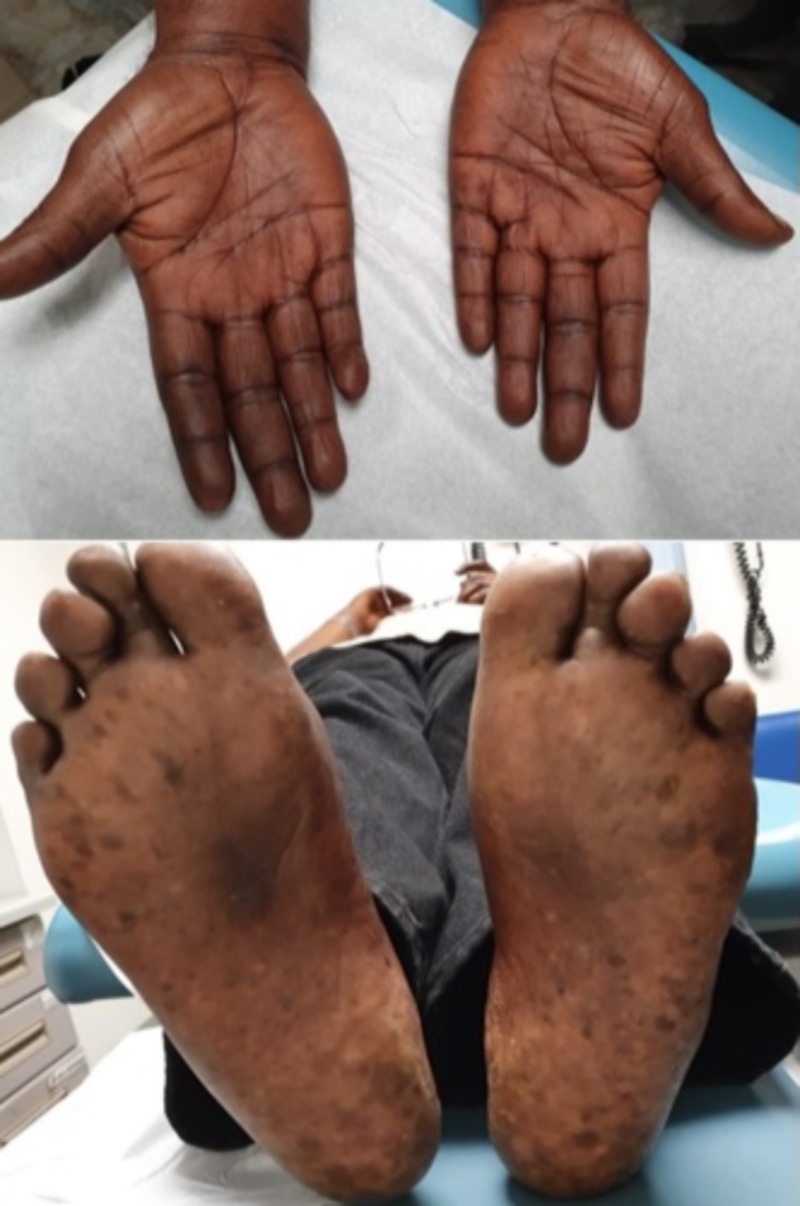
Hyperpigmentation of the palmar and plantar skin.

He had also noted darkening of the skin over the knuckle and the palms and soles as well (Figure 3). 

The nails were not thickened and brittle, and the surrounding skin was normal. There was no swelling, warmth, tenderness or any other abnormalities. The patient was concerned about the cosmetic appearance and chose to discontinue HU. The platelet count went back up to 850 K/µL. He was started on anagrelide with normalization of the platelet count.

## Discussion

ET is a BCR-ABL1 negative MPN associated with high platelet production leading to persistent thrombocytosis (platelet count ≥ 450 × 10^9^/L) and predisposition for vascular events [4]. Patients with ET have JAK2 mutation at 60%-65%, CALR mutation 20%-25%, MPL mutation 3%-5% or triple-negative 10% [5]. A study of 509 MPN patients with MPL mutation showed that 7% of them had a somatic mutation of MPL exon 10 [6]. A combination of HU and aspirin is the standard first-line treatment for high-risk and intermediate-risk ET [4]. The previous reports of HU-induced cutaneous toxicity are from older publications, and the driver mutations of ET are not well known. This is the first report of HU-induced cutaneous toxicity in an African American patient with ET with MPL mutation.

Several chemotherapeutic agents are known to cause mucocutaneous hyperpigmentation, most commonly cyclophosphamide, platinum agents and doxorubicin [7]. HU is a well-tolerated oral chemotherapeutic drug frequently used in the treatment of MPNs, including ET. It is an antimetabolite and exerts its antitumor activity by inhibiting the enzyme ribonucleotide reductase and thereby inhibiting DNA synthesis [1]. While HU is a well-tolerated agent, it is known to have skin toxicity such as skin ulceration, oral aphthosis and mucocutaneous dyschromia. A large review of HU-induced skin toxicity found non-ulcerative skin toxicity to occur in 1.6% of all patients [8]. It led to permanent discontinuation in 52% patients [8]. There have been two case series of patients with mucocutaneous hyperpigmentation [3,9]. This case is a unique example of one patient having palmoplantar, skin, nail and tongue (mucosal) discoloration.

The HU-induced nail discoloration may be transverse, longitudinal or diffuse, with longitudinal being the most common. Skin and palmoplantar discoloration is frequently seen with nail changes. The tongue discoloration is rare. The usual onset of discoloration is several months, and the temporal relation between start of HU and time of onset helps with diagnosis. While there is no known relationship between HU dose and hyperpigmentation, reports of tongue ulceration related to HU have been reported to occur with higher doses i.e., HU 3,000 mg per day [10]. A review of more than 3,000 patients with MPNs showed only less than 1% patients to have cutaneous dyschromia, and the median daily dose of HU was 1 gm at time of toxicity. The mean time on HU to toxicity was 60 months [11]. Interestingly, there was no difference in median dose in patients having skin ulceration, skin dyschromia or other mucosal adverse effects [11].

In a patient presenting with HU-related hyperpigmentation, it is important to rule out mimicking conditions such as subungal melanoma, especially in one nail involvement. Finger nails may have more hyperpigmentation then toenails due to slower growth of the nail, as was seen in our case as well. Discontinuation of HU frequently leads to slow improvement in the hyperpigmentation. 

The mechanism of HU-induced hyperpigmentation is not clearly understood, but it is hypothesized to be due to activation of melanocytes by HU which leads to increased melanin production which is then deposited in the nail bed [11]. Photosensitivity and genetic predisposition have been postulated to contribute as well [7,9].

## Conclusions

HU-induced cutaneous toxicity can be a source of anxiety for a patient and lead to medication discontinuation. Early and accurate recognition of this clinical condition can avoid unnecessary diagnostic interventions and, with simple change in medication, patients can be successfully treated. Patient education prior to starting HU can potentially reduce or mitigate anxiety.
